# Disease-Specific Anxiety in Chronic Obstructive Pulmonary Disease: Translation and Initial Validation of a Questionnaire

**DOI:** 10.3389/fpsyg.2022.907939

**Published:** 2022-07-05

**Authors:** Ingeborg Farver-Vestergaard, Sandra Rubio-Rask, Signe Timm, Camilla Fischer Christiansen, Ole Hilberg, Anders Løkke

**Affiliations:** ^1^Department of Medicine, Lillebaelt Hospital, Vejle, Denmark; ^2^Research Unit, Lillebaelt Hospital, Kolding, Denmark; ^3^Department of Regional Health Research, University of Southern Denmark, Odense, Denmark

**Keywords:** disease-specific fear, respiratory illness/disease, psychological distress, assessment, measurement, Danish translation

## Abstract

**Background:**

Commonly applied measures of symptoms of anxiety are not sensitive to disease-specific anxiety in patients with chronic obstructive pulmonary disease (COPD). There is a need for validated instruments measuring COPD-specific anxiety. Therefore, we translated the COPD-Anxiety Questionnaire (CAF) into Danish (CAF-R-DK) and performed an initial validation of the psychometric properties in a sample of patients with COPD.

**Materials and Methods:**

Translation procedures followed the World Health Organization guidelines. Participants with COPD completed questionnaires measuring COPD-specific anxiety (CAF-R-DK), general psychological distress (Hospital Anxiety and Depression Scale) as well as variables related to COPD (COPD Assessment Test; modified Medical Research Council dyspnea scale), quality of life (the 12-item Short Form survey, SF12), and socio-demography.

**Results:**

A total of 260 patients with COPD (mean age: 65.0, 69% female) completed questionnaires. The Danish version of CAF-R-DK demonstrated acceptable Cronbach’s α values that were comparable with those of the original CAF. As expected, the CAF-R-DK showed positive correlations with convergent constructs (CAT; HADS) and negative correlations with discriminant constructs (SF-12). However, the results for specific subdomains of the CAF-R-DK indicated inconsistency in the underlying concept of disease-specific anxiety, which was also suggested based on the subsequent confirmatory and exploratory factor analyzes.

**Conclusion:**

The CAF could serve as an important supplement to generic psychological distress screening of patients with COPD in somatic health care settings, and the questionnaire is now available in Danish. Translation into other languages is needed with the purpose of obtaining data for further testing the psychometric properties of the questionnaire.

## Introduction

Living with breathlessness, reduced mobility, and uncertainty related to chronic obstructive pulmonary disease (COPD) is associated with high levels of anxiety for many patients (Willgoss and Yohannes, 2013; von Leupoldt, 2017). Anxiety is a natural psychophysiological response to a perceived threat, which for most patients occur as passing emotional states in relation to external events (e.g., exacerbations of symptoms, hospitalization, or receiving messages with negative contents related to health and/or treatment options) or internal events (e.g., bodily sensations or intrusive thoughts or memories). However, for a considerable proportion of patients with COPD, symptoms of anxiety are persistent over a longer period of time and can be associated with behavioral avoidance of activities that are expected to trigger dyspnea (Holas et al., 2017; Stoeckel et al., 2018; Hanania and O’Donnell, 2019). Hence, symptoms of anxiety can aggravate daily functioning and quality of life, and are related to increased healthcare utilization, morbidity, and mortality in COPD (Eisner et al., 2010).

General symptoms of anxiety can be assessed with questionnaires such as the Beck Anxiety Inventory (BAI) (Fydrich et al., 1992), the General Anxiety Disorder-7 (GAD-7) (Spitzer et al., 2006), and the Hospital Anxiety and Depression Scale (HADS) (Zigmond and Snaith, 1983). In COPD, the HADS is most commonly applied (Smid et al., 2017b; Larsen et al., 2021), and was initially developed for use in somatic disease populations to avoid ceiling effects due to overlap between physical symptoms from the somatic disease and the psychological condition, respectively. Routine screening for general symptoms of anxiety and depression are important with the purpose of detecting potential psychological comorbidities in COPD, but the existing instruments has been criticized for suboptimal screening accuracy against diagnostic interviews (Baker et al., 2018). Moreover, recent studies differentiates general from disease-specific symptoms of anxiety in COPD, and stress the importance of assessing both aspects in COPD care (von Leupoldt and Janssens, 2016; Reijnders et al., 2019). Reijnders et al. (2019) showed that greater reductions in COPD-specific anxiety were associated with greater improvements in exercise capacity, quality of life, and health status as well as a more pronounced decrease in depression over the course of a pulmonary rehabilitation program – independent of general anxiety levels. Moreover, Keil et al. (2014) showed that COPD-specific anxiety contributed independently to disease-specific disability after statistically controlling for disease severity, sociodemographic variables, and general anxiety.

While several instruments measuring general symptoms of anxiety exist, fewer disease-specific anxiety measures are available for use in respiratory disease populations. The Anxiety Inventory for Respiratory Disease (Willgoss et al., 2013) was developed according to the same principles as the HADS, where somatic symptoms are excluded. This increases the specificity of the questionnaire, which means that the risk of identifying false positive cases is lowered, but at the same time it reduces the sensitivity, which leads to an increased risk of overseeing true positive cases (Trevethan, 2017; Baker et al., 2018). The Interpretation of Breathing Problems Questionnaire (Sutton et al., 1999) and the Breathlessness Catastrophizing Questionnaire (Solomon et al., 2015) are examples of questionnaires designed to measure dyspnea-related anxiety in respiratory illness, and they are therefore not sensitive toward other aspects of COPD-specific anxiety.

The COPD-Anxiety Questionnaire (CAF) was developed in Germany in 2011 to assess COPD-specific anxiety (Kühl et al., 2011). The original, German-language scale consists of 27 general items and 8 conditional items for patients with a partner and/or who receive oxygen treatment. The items cover the five subdomains of Fear of dyspnea (FD), Fear of physical activity (FPA, Fear of progression (FP), Fear of social exclusion (FSE), and Sleep-related worries (SRW). A shorted, 20-item version (CAF-R) was validated in 2014 (Keil et al., 2014). While questionnaires assessing general symptoms of anxiety have long been available in multiple languages, measurement instruments of disease-specific anxiety were developed relatively recently, and have therefore not yet been translated and validated more broadly.

On this background, the present study aimed to translate the CAF-R into Danish, and to validate the Danish version of the questionnaire (CAF-R-DK) in a sample of patients with COPD, including an initial evaluation of different response formats, i.e., electronic and paper-version.

## Materials and Methods

The translation and initial validation strategy was based on the World Health Organization’s (WHO) guidelines for the process of translation and adaptation of instruments^1^ and is described in detail below.

### Translation Procedures

The initial translation of the CAF from German into Danish language was performed by the second author (SRR) who is a nurse with considerable experience within respiratory medicine. The translator is knowledgeable of the German-speaking culture and her mother tongue is Danish. The initial translation was based on a conceptual rather than literal approach, aiming at the conceptual equivalent of a given word or phrase rather than a word-by-word translation. Use of technical terms, colloquialism, idioms, or vernacular terms was avoided. Back-translation was performed by two independent native German translators, who had no prior knowledge of the questionnaire. Discrepancies were discussed until an agreement was reached.

An expert panel of researchers from different relevant disciplines (i.e., psychology, respiratory medicine, pulmonary rehabilitation) discussed the Danish translation of the CAF-R. Relevant adjustments of the CAF-R-DK were made on the basis of the suggestions from the panel.

### Pre-Testing and Cognitive Interviewing

Five pre-test respondents with a diagnosis of COPD were recruited from the pulmonary outpatient clinic at the Department of Medicine, Lillebaelt Hospital, Vejle, Denmark. Each respondent completed the questionnaire individually and immediately thereafter participated in a debriefing asking (1) whether they could repeat the question in their own words, (2) what came to their mind when they heard a particular phrase or term, and (3) explain how they choose their answer. The questions were repeated for each item in the questionnaire. Hereafter, the cognitive interviewing was based on understanding potential conflicts in verbal answers and questionnaire-answers with the purpose of reaching consistency. Relevant adjustments of the CAF-R-DK were made on the basis of the interview material to ensure face validity.

### Questionnaires

A questionnaire package was distributed electronically via the webpage and social media platforms of the Danish Lung Association. Furthermore, a paper-version of the questionnaire package was distributed among COPD outpatients at the Department of Medicine, Lillebaelt Hospital, Vejle, Denmark. The questionnaire package consisted of the following questionnaires.

*The Danish version of the COPD-Anxiety Questionnaire 20-item version (CAF-R-DK)* (Keil et al., 2014) was applied to measure COPD-specific anxiety. The questionnaire consists of 20 item each rated on a Likert scale from 0 = “never” to 4 = “always.” The items cover the domains of Fear of dyspnea (“When I become short of breath, I get scared”), Fear of physical activity (“I avoid physical exertion”), Fear of progression (“I fear that someday I will become a burden for others because of my illness”), Fear of social exclusion (“I feel left alone with my illness”), and Sleep-related worries (“I wake up at night because of my breathing”). The original CAF-questionnaire has good internal consistency, with Cronbach’s α of domain scales ranging from α = 0.78 (SRW) to α = 0.87 (FSE, FP) (Keil et al., 2014). The Danish version of the questionnaire can be obtained from the authors upon request.

*The COPD Assessment Test (CAT)* (Jones et al., 2009) was included with the purpose of measuring COPD-specific disability, or health status, and consists of 8 items that are rated on a semantic differential scale from 0 (e.g., “I am not limited doing any activities at home”) to 5 (e.g., “I am very limited doing activities at home”). The CAT is commonly applied in COPD research and clinical practice and shows good psychometric properties, e.g., Cronbach’s α = 0.88 (Jones et al., 2009, 2011; Kon et al., 2014; Smid et al., 2017a).

*The modified Medical Research Council dyspnea scale (mMRC)* (Williams, 2017) was applied to measure the degree of dyspnea on a Likert scale from 0 = “I only get breathless with strenuous exercise” to 4 = “I am too breathless to leave the house or I am breathless when getting dressed.” The scale is recommended for baseline assessment of dyspnea in the Global Initiative for Chronic Obstructive Lung Disease (GOLD) guidelines (GOLD, 2017).

*The Hospital Anxiety and Depression Scale (HADS)* was used to measure symptoms of anxiety and depression. The scale consists of 14 items, including 7 for symptoms of depression (e.g., “I feel as if I am slowed down”) and 7 for general symptoms of anxiety (e.g., “I feel tense or ‘wound up”). Items are rated on a semantic differential scale from 0 (e.g., “Not at all”) to 3 (e.g., “Most of the time”). The scale is commonly used as a screening tool in COPD, with a Cronbach’s α of 0.87 (Baker et al., 2018).

*The 12-item Short Form survey (SF-12)* (Ware et al., 1996) was applied to measure quality of life by addressing different aspects of emotional states and daily activities (e.g., “Have you felt down-hearted and blue?”; “Have you accomplished less than you would like as a result of your physical health?”). Total scores for the physical components score (PCS) and the mental component score (MCS), respectively, are calculated based on population norms (score range from 0–100), with higher scores indicating better health. The SF-12 demonstrates good sensitivity to change and discriminative values in grades of COPD (Menn et al., 2010).

### Analysis

All analyzes were performed using Stata 17 software (StataCorp. 2021. *Stata Statistical Software: Release 17*. College Station, TX, United States: StataCorp. LLC). For all analyzes, *p*-values of ≤ 0.05 were considered statistically significant. Correlation coefficients of 0.20, 0.40, 0.60, and 0.80 were considered weak, moderate, strong, and very strong, respectively. Missing data were imputed by mean substitution, except from the SF-12, where missing data were handled according to the scoring descriptions (Ware et al., 1995) (the full description can be found in Supplementary Material 1). For the CAF-R-DK, missing data on individual items were explored with the purpose of identifying whether particular items in the CAF-R-DK indicated problems in understanding the content (Primdahl et al., 2021).

Convergent validity was tested with Spearman’s rank correlation coefficients. We expected positive correlations with all convergent variables (i.e., CAT, HADS-Anxiety, and HADS-Depression) and negative correlations with discriminant variables (i.e., SF-12 PCS and SF-12 MCS). Reliability was assessed by computing Cronbach’s α. A confirmatory factor analysis was conducted to evaluate whether the factor structure of the CAF-R-DK corresponded to the original CAF-R. If a less than acceptable fit was obtained, a supplementary exploratory factor analysis was performed to evaluate the number of latent variables/subdomains of the CAF-R-DK, and whether individual items belonged to subdomains other than those hypothesized by the original CAF-R. Bartlett’s test of sphericity (Bartlett, 1951) and Kaiser-Meyer-Olkin (KMO) sampling adequacy (Kaiser, 1974) were calculated to ensure that factor analysis was appropriate.

## Results

In the period from May to September 2020 a total of 260 participants completed the questionnaire package. Respondents for electronic and paper-based completion were recruited in parallel. A total number of 333 individuals opened the link to the online questionnaire, resulting in 238 (71.5%) completed electronic responses. The remaining responses (*n* = 22) were paper-based. See Table 1 for an overview of participant characteristics.

**TABLE 1:** Participant characteristics.

Total sample (*n* = 260)	Mean	SD	*N*	%
Age	65.0	9.0		
Female gender			179	68.8
**Obstruction (FEV1% pred.)**				
Mild (>80%)			8	3.1
Moderate (50–80%)			62	23.8
Severe (30–50%)			85	32.7
Very severe (<30%)			89	34.2
**Smoking status**				
Current smoker			36	13.8
Previous smoker			196	75.4
Never smoker			9	3.5
Living alone (vs. living with a partner)			115	44.2
LTOT users			39	15.0
mMRC dyspnea	2.3	1.1		
CAT total	19.6	7.2		
HADS total	14.0	7.9		
SF-12 PCS	30.0	7.2		
SF-12 MCS	43.9	12.8		
CAF-R-DK-total	39.7	15.4		
CAF-R-DK-FSE	8.0	3.9		
CAF-R-DK-FD	9.1	4.4		
CAF-R-DK-FPA	10.2	5.2		
CAF-R-DK-FP	10.2	4.1		
CAF-R-DK-SRW	2.2	1.8		

*CAF-R, COPD anxiety questionnaire revised; CAT, COPD assessment test; FEV1% pred, forced expiratory volume in 1 second,% of predicted value; FD, fear of dyspnea; FP, fear of progression; FPA, fear of physical activity; FSE, fear of social exclusion; HADS, hospital anxiety and depression scale, range 0–42, higher score means more psychological distress; LTOT, long term oxygen therapy; mMRC, modified medical research council dyspnea scale, range 0–4, higher score means more dyspnea; SF-12, 12-item short form survey; PCS, physical component score; MCS, mental component score, range 0–100; higher score means; SRW, sleep-related worries.*

### Reliability and Construct Validity

Correlation coefficients of all validity and reliability analyzes can be found in Table 2. The CAF-R-DK total score and all individual domain scores showed acceptable to excellent reliability with Cronbach’s α ranging from 0.77 to 0.89. All convergent and discriminant construct showed the expected direction of the correlations (positive correlations for convergent constructs versus negative correlations for discriminant constructs). Correlations with convergent constructs were all moderate to strong (*r* = 0.39–0.69). The discriminant construct of SF-12 PCS was weakly correlated with the CAF, compared to the SF-12 MCS (PCS *r* = −0.04 to −0.30; MCS *r* = −0.43 to −0.67).

**TABLE 2:** Validity and reliability of the chronic obstructive pulmonary disease-anxiety questionnaire revised – Danish version (CAF-R-DK).

	CAF-R-DK FSE	CAF-R-DK FD	CAF-R-DK FPA	CAF-R-DK FP	CAF-R-DK SRW	CAF-R-DK-total
**Validity (convergent constructs)**						

CAT	0.44	0.56	0.60	0.46	0.62	0.67
HADS-Anx	0.46	0.60	0.43	0.55	0.43	0.62
HADS-Dep	0.57	0.53	0.59	0.55	0.39	0.69

**Validity (discriminant constructs)**						

SF-12 PCS	–0.12	–0.07	–0.30	–0.11	–0.04	–0.18
SF-12 MCS	–0.52	–0.55	–0.53	–0.56	–0.43	–0.67

**Reliability (internal consistency)**						

Cronbach’s α	0.87	0.85	0.89	0.87	0.77	−

*CAF-R-DK, COPD anxiety questionnaire revised – Danish version; CAT, COPD assessment test; FD, fear of dyspnea; FP, fear of progression; FPA, fear of physical activity; FSE, fear of social exclusion; HADS, hospital anxiety and depression scale; SF-12, 12-item short form survey; PCS, physical component score; MCS, mental component score; SRW, sleep-related worries.*

### Internal Consistency

The p-value for Bartlett’s test of sphericity was < 0.001 and the KMO measure of sampling adequacy was 0.907, which were both sufficient for conducting factor analysis (Bartlett, 1951; Kaiser, 1974).

A confirmatory factor analysis was conducted to test whether the structure of the CAF-R-DK corresponded to the original version of the questionnaire. The root mean square error of approximation was 0.087 (CIs: 0.077–0.097; *p* = 0.000), the Comparative Fit Index was 0.911, and the Tucker-Lewis index was 0.894, which altogether indicate a less than acceptable fit (Fan et al., 1999).

Hence, a polychoric exploratory factor analysis with oblique rotation was conducted with the purpose of exploring whether the number of latent variables in the CAF-R-DK was different from those identified in the original version of questionnaire, or whether the individual items loaded on different latent variables than described. Horn’s parallel analysis (Dinno, 2009), together with a scree plot, was conducted with the purpose of assessing the number of latent variables, and the existence of five factors in the questionnaire was confirmed (see graph in Supplementary Material 2). Table 3 shows the factor loadings for each individual item in the CAF-R-DK. For factors 2–5, the loading of the individual items of the CAF-R-DK were comparable to those of the original questionnaire. However, items originally belonging to the subscales of Fear of dyspnea (e.g., “When I get short of breath I am afraid that I will suffocate”), Fear of physical activity (i.e., “I plan the route in detail before I go for a walk”), and Fear of progression (i.e., “I am afraid that my breathing problems will become worse”) all loaded on the same factor (factor 1) in the present dataset.

**TABLE 3:** Factor loadings of items from the chronic obstructive pulmonary disease-anxiety questionnaire revised – Danish version (CAF-R-DK).

Item	Subscale in original questionnaire	Factor 1	Factor 2	Factor 3	Factor 4	Factor 5	Missing n (%)
1	SRW	−0.0078	0.0014	0.0133	0.0643	**0.7767**	19 (7.3)
2	FPA	0.0144	**0.8965**	−0.0285	−0.1335	0.0825	17 (6.5)
3	FSE	0.0958	0.0121	**0.8594**	−0.1324	−0.0404	17 (6.5)
4	FD	**0.8300**	−0.0087	−0.0599	−0.0802	0.2312	18 (6.9)
5	FPA	**0.3667**	**0.3667**	−0.0910	0.1400	0.1109	18 (6.9)
6	FP	0.0836	0.0914	−0.0287	**0.7065**	0.0862	19 (7.3)
7	FSE	−0.0976	0.2109	**0.6512**	0.0692	0.0286	16 (6.2)
8	FPA	−0.0432	**0.8412**	0.0974	0.0363	0.0421	16 (6.2)
9	FD	**0.4163**	−0.0644	0.1889	−0.1005	0.3174	17 (6.5)
10	FP	**0.5491**	0.1275	0.1424	0.2417	−0.3232	17 (6.5)
11	FD	**0.4736**	0.0039	0.1642	0.0477	0.1246	19 (7.3)
12	FSE	−0.0990	−0.0564	**0.8428**	0.1408	0.0944	17 (6.5)
13	FD	**0.8351**	−0.0254	0.0315	0.0809	0.0369	19 (7.3)
14	FSE	0.0850	0.0116	**0.8162**	0.1009	−0.0651	19 (7.3)
15	FPA	0.0242	**0.9205**	0.0668	−0.0362	−0.0712	20 (7.7)
16	SRW	−0.0597	0.0793	−0.0285	0.0459	**0.8131**	19 (7.3)
17	FP	−0.0061	−0.0846	0.0736	**0.9066**	0.1077	20 (7.7)
18	FP	0.0625	−0.0233	0.0207	**0.9034**	−0.0212	21 (8.1)
19	FPA	0.0087	**0.9276**	−0.0652	0.0551	0.0290	20 (7.7)
20	FD	**0.8509**	0.0664	−0.0017	0.1383	−0.1764	20 (7.7)

*CAF-R, COPD anxiety questionnaire revised; CAT, COPD assessment test; FD, fear of dyspnea; FP, fear of progression; FPA, fear of physical activity; FSE, fear of social exclusion; SRW, sleep-related worries.Notes: The highest factor loading for each item on the CAF-R-DK, thus identifying a factor, is marked in bold.*

### Missing Values

An overview of missing values per item can be found in Table 3. Number of missing values for each item in the CAF-R-DK varied from 16 (6.2%) on Item 8 (“I avoid activities that make me sweat”) to 21 (8.1%) on Item 18 (“I fear that I will eventually become dependent of care from others because of my illness”). Compared to electronic questionnaire responses, paper-based responses had a very low number of missing items, with only one participant missing one item (Item 1).

## Discussion

It has been proposed that many patients with COPD are living with unrecognized symptoms of anxiety due to the poor availability of instruments measuring disease-specific anxiety in this population (Yohannes and Lavoie, 2013; Breland et al., 2015; Larsen et al., 2021). The COPD Anxiety Questionnaire (CAF) is an example of a COPD-specific anxiety instrument that has been developed in German language, but is still not available in multiple languages. For the purpose of the present study, we translated the short version of the CAF (CAF-R) into Danish (CAF-R-DK) and performed an initial validation of its psychometric properties in a sample of 260 patients with COPD across all degrees of obstructive lung function impairment (mild, moderate, severe, and very severe).

The results of the present study indicated that the CAF-R-DK showed acceptable to excellent reliability with Cronbach’s α scores of subscales ranging from 0.77 to 0.89, which are also comparable with reliability scores of the original questionnaire (Cronbach’s α of subscales: 0.78–0.87 (Keil et al., 2014).

With respect to construct validity, the CAF-R-DK showed positive correlations with converging constructs [COPD-symptoms level (CAT), general symptoms of anxiety (HADS-Anxiety), symptoms of depression (HADS-Depression)] and negative correlations with discriminant constructs [physical quality of life (SF-12 PCS), mental quality of life (SF-12 MCS)], as expected. Concerning the individual converging constructs, the total score of the CAF-R-DK showed higher correlations with the HADS-depression scale (*r* = 0.69), compared to the HADS-anxiety scale (*r* = 0.62). This is surprising, as the construct of disease-specific anxiety was expected to correlate relatively more with general symptoms of anxiety, compared with symptoms of depression. A possible explanation could be that the correlation with symptoms of depression are driven by the specific CAF-subdomains Fear of social exclusion (*r* = 0.57) and Fear of physical activity (*r* = 0.59), which include items such as “I feel let alone with my illness” and “I avoid all kinds of physical activity”. Such items could be interpreted as expressions of hopelessness and lethargy, respectively, which are characteristic of depressive states (Osler, 2021). Moreover, the converging construct of COPD symptoms, measured with the CAT, also showed a relatively higher correlation (*r* = 0.67) with the CAF-R-DK total score, than the HADS-Anxiety. This was especially the case for the CAF-subdomains of Fear of physical activity (*r* = 0.60) and Sleep-related worries (*r* = 0.62), the latter consisting of items such as “The sound of my breathing or coughing wake me up at night”. The wording of such items could be more indicative of physical symptom level than of anxious symptom interpretation, which may explain the higher correlation of the CAF-R-DK with COPD symptom level than with general symptoms of anxiety. On the other hand, when inspecting correlations of the CAF-R-DK with the discriminant constructs of physical and mental quality of life, all subdomains of the CAF-R-DK showed considerably stronger correlations with the mental component score of the SF-12 (*r* = −0.67), compared with the physical component score (*r* = −0.18). This finding supports CAF-R-DK as measuring the predominantly psychological, not physical, construct of disease-specific anxiety. Taken together, the present study results indicate that disease-specific anxiety in COPD can be understood as a psychophysiological construct, including and correlating strongly with physical symptoms and sensations (O’Donnell et al., 2007; Ora et al., 2010; Hanania and O’Donnell, 2019). However, the results could also be indicative of relatively poor construct validity and wording of individual items in the CAF, which should be kept in mind when applying the questionnaire and interpreting its results in future studies and clinical practice. As the present study did not perform a direct comparison between the original version and the Danish translation of the questionnaire, it is unknown whether these findings are true only for the CAF-R-DK, or whether they could be extrapolated to the CAF more generally. However, the developers of the original version of the CAF (Kühl et al., 2011) appear to have collected relatively limited data for the conceptualization of disease-specific anxiety prior to designing the questionnaire, i.e., five patient interviews and items from the Cardiac Anxiety Questionnaire (Eifert et al., 2000), which may have compromised the construct validity of the questionnaire. In addition to cardiac-related anxiety, the construct of disease-specific anxiety in COPD may also share features with anxiety in asthma, measured with the Asthma-Related Anxiety Scale (Bruzzese et al., 2011). Moreover, in other to determine the specific characteristics of anxiety in COPD in the future, a mapping of shared and distinctive features of anxiety in COPD, asthma, cardiac disease, multiple sclerosis and other diseases that are characterized by chronic impairment and a high risk of acute symptom worsening (i.e., exacerbations; attacks) is needed (Murray et al., 2005).

Furthermore, concerning the internal consistency of the CAF-R-DK, the initial confirmatory factor analysis of the structure of the original version of the questionnaire did not result in a good fit to the data in the present study. In the subsequent exploratory factor analysis, the five-factor structure of the questionnaire was confirmed, but individual items that belonged to the Fear of physical activity (i.e., “I plan the route in detail before I go for a walk”) and Fear of disease progression (i.e., “I am afraid that my breathing problems will become worse”) subscales in the original version of the questionnaire loaded on the Fear of dyspnea scale (e.g., “When I get short of breath I am afraid that I will suffocate”) in the present study. The results do not allow for any conclusions as to whether these differences stem from language- or cultural differences between the German and Danish version of the questionnaire or whether they are resulting from a suboptimal scale construction of the original version of the CAF. However, researchers and clinicians should be aware that it can be difficult to differentiate certain latent variables of disease-specific anxiety, and there may be significant overlap between the experience of fear of dyspnea, fear of physical activity, and fear of disease progression.

When inspecting the number of missing answers for the items in CAF-R-DK, there was a relatively high proportion of missing values across all items (6.2–8.1%). As 16 respondents (6.2%) missed the entire CAF-R-DK, the missing responses are less likely to be a result of poor understanding of individual items. Missing values were predominantly observed for the electronic responses, while only one participant missed one item in the paper-based responses. This may speak to an increased feasibility of the paper-version of the CAF-R, but it also stands in contrast to other studies, showing a higher number of missing in paper-based responses (Palen et al., 2008; Shih and Fan, 2009), and the feasibility of electronic vs. paper-based questionnaire formats appears to depend on the specific respondent population (Shih and Fan, 2009). Hence, the relatively high missing rates in the electronic responses of the present study should not prevent researchers from using the electronic format in future studies. However, “forced responding” in the electronic version of the CAF could be considered (Nayak and Narayan, 2019).

### Clinical Implications

The results of the present study expand the availability of instruments for the assessment of disease-specific anxiety in COPD, which is relevant for psychological screening in routine care, e.g., rehabilitation clinics, general practice, and outpatient hospital visits. In a study by Hardy et al. (2014), primary care nurses were trained in following a systematic psychological screening and intervention pathway during the annual review of 35 patients with COPD. The results of their evaluation showed that 75% of the patients felt pleased about being asked questions regarding depression and anxiety, while the remaining patients reported that they had no particular feelings. Moreover, 91% reported that they felt more motivated to manage their symptoms after the screening and consultation with the nurse. On the other hand, screening and assessment of anxiety in clinical practice can be obstructed by certain barriers among healthcare professionals. In an editorial, Heslop-Marshall and Burns (2019) present three important barriers: (1) Clinicians may not recognize the scale of the problem, assuming that the symptoms of anxiety is a natural part of the ‘psychological makeup’ of the patient with COPD; (2) Clinicians may consider psychological symptoms to be outside their professional remit; (3) Access to appropriate therapy and availability of trained therapists are scarce, and clinicians may therefore consider identification of psychological symptoms pointless. The results of the present study do not allow for conclusions it terms of (barriers to) the practical use of the CAF-R-DK, and it is therefore important to address such barriers in future research with the purpose of achieving and optimal implementation of psychological screening procedures in clinical practice.

Moreover, when applying instruments such as the CAF, clinicians should be aware that a high CAF-score is not necessarily equal to high level of general symptoms of anxiety or to the presence of a mental *disorder*, e.g., panic disorder, generalized anxiety disorder, social phobia, and post-traumatic stress disorder, for all of which a high prevalence rate has been demonstrated in COPD (Yohannes et al., 2010; Willgoss and Yohannes, 2013; Ouellette and Lavoie, 2017). Disease-specific anxiety and accompanying, maladaptive avoidance or safety behaviors could potentially increase the *risk* of developing an anxiety disorder, but future studies, including diagnostic interviews, are needed to confirm the relationship between disease-specific anxiety and the development of anxiety disorders and other mental disorders in COPD.

### Strengths and Limitations

The present study has several strengths. First, owing to the electronic participation option, the study is based on a large sample of patients with COPD with all degrees of obstructive lung function impairment (mild, moderate, severe, and very severe), including a considerable proportion of long-term oxygen therapy users (15%) who can be difficult to reach for research purposes. Second, the translation of the CAF-R into Danish is based on a comprehensive and systematic approach, which ensures a high-quality version of the questionnaire ready for application in the Nordic countries. Third, taking a comprehensive approach to measurement, the questionnaire package of the present study includes several relevant scales that allows for assessment of associations with both convergent and discriminant constructs.

However, a number of limitations should also be noted. First, the questionnaire format, i.e., electronically versus paper-based, were applied in two different recruitment setups, i.e., patient organization versus outpatient clinic, and a direct comparison of the response formats could therefore not be performed. Studies using random assignment to electronic versus paper versions of the questionnaire are needed to test whether the response format impacts the validity of the questionnaire. Second, due to recruitment anonymity for online respondents, and with the purpose of limiting participation burden for patients in the present study, the single measurement design did not allow for assessment of test-retest reliability of the questionnaire. A more comprehensive testing of the psychometric properties of the CAF-R-DK is needed to confirm longitudinal, predictive validity and reliability. Third, while the CAF is currently the only existing instrument measuring COPD-specific anxiety beyond fear of dyspnea, the quality of the questionnaire can be criticized: (a) The initial conceptualization of the construct of COPD-specific anxiety was based on five patient interviews (Kühl et al., 2011), which can be considered as a relatively restricted database (de Vet et al., 2011). (b) The developers of the questionnaire (Kühl et al., 2011) claim that the wording of the items was based on the Cardiac Anxiety Questionnaire (Eifert et al., 2000). But very few similarities between the wording of the two questionnaires can be found, and several items of the CAF does not adhere to suggested standards for item wording (e.g., avoiding negative wording; items should be specific) (de Vet et al., 2011). (c) The questionnaire has not been translated into English, leading to relatively limited application and testing of the questionnaire worldwide. Lastly, due to the experiential nature of the construct of anxiety, it is not possible to validate the scale against a “gold standard,” objective measurement method, which prevents the analyzes of predictive validity as well as the determination of a clinically significant cut-point. Future studies could benefit from performing a direct comparison between CAF scores and diagnostic interviewing by a mental health specialist.

## Conclusion

A Danish version of the COPD-Anxiety Questionnaire (CAF-R-DK) is now available for the assessment of disease-specific anxiety in Danish-speaking patients with COPD. The CAF should not be used as an alternative to screening for symptoms of anxiety in general, but can be applied as an important supplement to the Hospital Anxiety and Depression Scale (HADS) (as an example) with the purpose of identifying relevant areas of disease-specific anxiety that might act as barriers for outcomes of rehabilitation programs. There might be general inconsistencies in the construct validity of the CAF-R-DK and/or the CAF in general. In the future, there is a need for translation of the questionnaire into other languages with the purpose of obtaining clinical and research-based data on the psychometric properties and practical application of the CAF. Moreover, there is a need for studies that aim to test measures of disease-specific anxiety against general anxiety questionnaires and diagnostic interviews in COPD.

## Data Availability Statement

The raw data supporting the conclusions of this article will be made available by the authors, without undue reservation.

## Ethics Statement

Ethical review and approval was not required for the study on human participants in accordance with the local legislation and institutional requirements. Written informed consent for participation was not required for this study in accordance with the national legislation and the institutional requirements.

## Author Contributions

IF-V had a leading role in the conception and design of the present study as well as in the data analysis and drafting of the present manuscript. SR-R had a leading role in data acquisition. ST contributed to the statistical analysis. SR-R, ST, CFC, OH, and AL contributed to the conception and interpretation of data for the work and to revising the contents of the present manuscript. All authors provided approval for publication of the content of the present manuscript and agreed to be accountable for all aspects of the work in ensuring that questions related to the accuracy or integrity of any part of the work are appropriately investigated and resolved.

## Conflict of Interest

The authors declare that the research was conducted in the absence of any commercial or financial relationships that could be construed as a potential conflict of interest.

## Publisher’s Note

All claims expressed in this article are solely those of the authors and do not necessarily represent those of their affiliated organizations, or those of the publisher, the editors and the reviewers. Any product that may be evaluated in this article, or claim that may be made by its manufacturer, is not guaranteed or endorsed by the publisher.

## Footnotes

1
https://cdn.who.int/media/docs/default-source/publishing-policies/whoqol-100-guidelines/translation-methodology.pdf?sfvrsn=74cdb8f5_2
