# Supplementary material for: Disease-Specific Anxiety in Chronic Obstructive Pulmonary Disease: Translation and Initial Validation of a Questionnaire
Source: Front Psychol. 2022 Jul 5;13:907939. doi: 10.3389/fpsyg.2022.907939 (PMC9294537; doi:10.3389/fpsyg.2022.907939)
Supplement: Supplementary file 1 [file Data_Sheet_1.pdf]

# *Supplementary Material*

## 1 Imputation of missing data in SF-12

Based on Hopman WM, Harrison MB, Carley M, et al. Additional Support for Simple Imputation of Missing Quality of Life Data in Nursing Research. *ISRN Nurs* 2011; 2011: 752320.

### 1.1 Introduction

This supplementary material describe the imputation strategy for replacing missing values with the mean of the population under study when less than half (≤5) of the required items of the SF-12 are missing. This approach is not suitable for cases where the entire questionnaire is missing or where there is a clear pattern of missing data.

### 1.2 Description of participants (completed vs. missing)

Complete SF-12 data were available for 198 (76.2%) of the 260 participants. Forty-three (16.5%) were missing one or more items. Finally, 19 (7.3%) missed the entire questionnaire and were therefore not included in the imputation.

Characteristics of participants who completed all items and participants eligible for imputation, respectively, can be found in Table 1.

#### 1.2.1 Table 1. Characteristics of participants who completed all items vs. missed 1-3 items on the SF-12

| | Completed all items | | | Eligible for imputation (missing 1-3 items) | | | Difference |
|---|---|---|---|---|---|---|---|
| | N | Mean | SD | N | Mean | SD | *p* |
| Age | 173 | 64.2 | 8.7 | 43 | 64.8 | 10.1 | 0.754 |
| Gender | | | | | | | 0.577 |
| Female | 143 | | | 33 | | | |
| Male | 54 | | | 10 | | | |
| Living situation | | | | | | | 0.350 |
| With partner | 99 | | | 24 | | | |
| Alone | 97 | | | 17 | | | |
| LTOT status | | | | | | | 0.148 |
| User | 29 | | | 10 | | | |
| Non-user | 168 | | | 32 | | | |
| MRC dyspnea | 198 | 3.2 | 1.1 | 43 | 3.5 | 1.1 | 0.156 |
| CAT total | 198 | 19.4 | 7.4 | 43 | 20.0 | 6.1 | 0.617 |
| HADS-total | 198 | 13.8 | 7.9 | 43 | 14.2 | 7.1 | 0.786 |
| CAF-DK-total | 194 | 39.0 | 15.5 | 42 | 41.7 | 14.2 | 0.301 |
| CAF-DK-FSE | 198 | 7.8 | 3.8 | 43 | 8.7 | 3.8 | 0.159 |
| CAF-DK-FD | 198 | 9.0 | 4.3 | 43 | 9.2 | 4.8 | 0.789 |
| CAF-DK-FPA | 198 | 10.0 | 5.2 | 43 | 11.0 | 4.9 | 0.261 |

| | | | | | | | |
|---|---|---|---|---|---|---|---|
| CAF-DK-FP | 198 | 10.1 | 4.1 | 43 | 10.6 | 3.8 | 0.482 |
| CAF-DK-SRW | 194 | 2.2 | 1.8 | 42 | 2.4 | 2.1 | 0.551 |

[a] Independent samples *t* test for continuous variables, chi$^2$ test for categorical variables

## 1.3 Frequency of missing items

For the 43 respondents that missed one or more items, 34 (13.1%) missed one item, 6 (2.3%) missed two items and three (1.2%) missed three items, resulting in 55 missing items. Of these, most where in the physically oriented items (37 (67%)) (See Table 2).

### 1.3.1 Table 2. Frequency of missing items on the SF-12

| SF-12 item | Domain | Component | Missing |
|---|---|---|---|
| 1 | General health | PCS | 2 |
| 2 | Physical function | PCS | 10 |
| 3 | Physical function | PCS | 4 |
| 4 | Role physical | PCS | 1 |
| 5 | Role physical | PCS | 8 |
| 6 | Role emotional | MCS | 4 |
| 7 | Role emotional | MCS | 5 |
| 8 | Bodily pain | PCS | 12 |
| 9 | Mental health | MCS | 2 |
| 10 | Vitality | MCS | 1 |
| 11 | Mental health | MCS | 3 |
| 12 | Social functioning | MCS | 3 |

## 1.4 Item means

Items 2, 5 and 8 all had a relatively high number of missings (>7). However, means and ranges of ratings on these items did not substantially differ in the group of participants who completed the questionnaire versus the group of participants who were eligible for imputation (See Table 3).

### 1.4.1 Table 3. Individual SF-12 item scores of participants who completed all items vs. missed 1-3 items

| Item | Completed all items | | | Eligible for imputation (missing 1-3 items) | | |
|---|---|---|---|---|---|---|
| | n | Mean score | Score range | n | Mean score | Score range |
| 1 | 198 | 3.9 | 1-5 | 41 | 3.8 | 1-5 |
| 2 | 198 | 1.6 | 1-3 | 33 | 1.3 | 1-3 |
| 3 | 198 | 1.4 | 1-3 | 39 | 1.5 | 1-3 |
| 4 | 198 | 2.7 | 1-5 | 42 | 2.4 | 1-5 |
| 5 | 198 | 2.6 | 1-5 | 35 | 2.5 | 1-5 |
| 6 | 198 | 2.9 | 1-5 | 39 | 2.8 | 1-5 |
| 7 | 198 | 3.3 | 1-5 | 38 | 3.2 | 1-5 |
| 8 | 198 | 3.6 | 1-5 | 31 | 3.4 | 1-5 |
| 9 | 198 | 2.7 | 1-5 | 41 | 2.8 | 1-5 |

| | | | | | | |
|---|---|---|---|---|---|---|
| 10 | 198 | 3.7 | 1-5 | 42 | 3.7 | 1-5 |
| 11 | 198 | 3.6 | 2-5 | 40 | 3.6 | 2-5 |
| 12 | 198 | 3.1 | 1-5 | 40 | 2.8 | 1-5 |

## 1.5 Post-imputation results

When the imputed data were added into the larger sample, the differences between mean PCS and MCS scores of participants who completed all items versus participants who had some of their data imputed was statistically non-significant, suggesting that the imputation had no significant impact on the data (See Table 4).

### 1.5.1 Table 4. SF-12 Component scores of participants who completed all items vs. missed 1-3 items

| Component | Completed all items (not imputed) | | | Missed 1-3 items (imputed) | | | Difference |
|---|---|---|---|---|---|---|---|
| | n | Mean | SD | n | Mean | SD | $p^a$ |
| PCS | 198 | 30.0 | 7.2 | 43 | 29.8 | 6.1 | 0.855 |
| MCS | 198 | 43.9 | 12.8 | 43 | 43.0 | 9.9 | 0.681 |

[a] Independent samples *t* test

## 2 Horn's parallel analysis with scree plot – CAF-R-DK

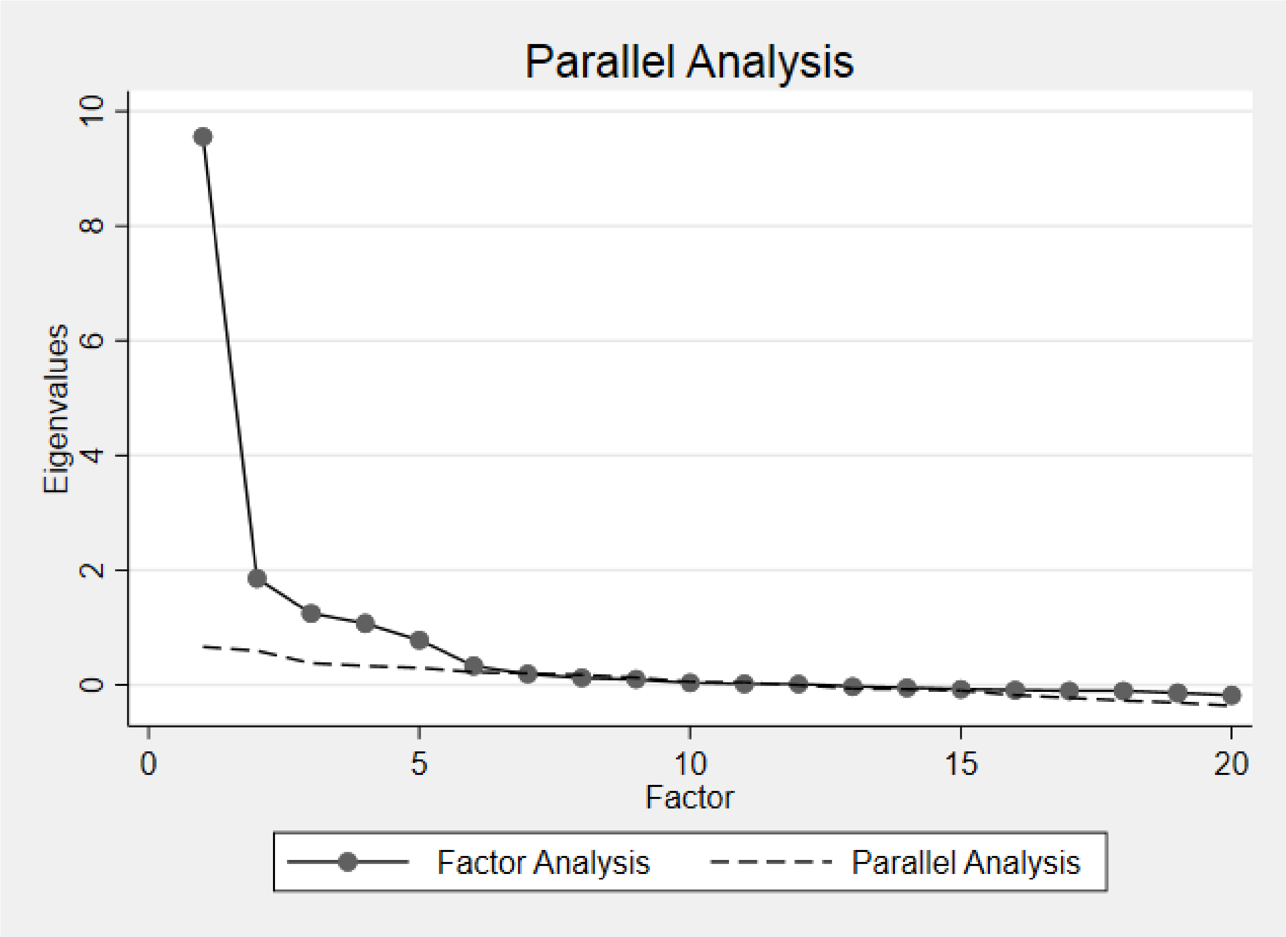


**Supplementary Figure 1.** Horn's parallel analysis with scree plot
